# Evolving Cystic Fibrosis Therapy: The Good, the Sad, and the Hopeful

**DOI:** 10.3390/children13070878

**Published:** 2026-06-30

**Authors:** Dominik Funken, Hartmut Grasemann

**Affiliations:** 1 Division of Respiratory Medicine, Department of Paediatrics, The Hospital for Sick Children, Toronto, ON M5G 1X8, Canada; dominik.funken@sickkids.ca; 2 Program in Translational Medicine, The Hospital for Sick Children, Toronto, ON M5G 1X8, Canada; 3 Institute of Medical Science Temerty, Faculty of Medicine, University of Toronto, Toronto, ON M5S 3H2, Canada

**Keywords:** cystic fibrosis, CFTR modulators, global health equity, genetic epidemiology, HEMT, low- and middle-income countries, newborn screening, diagnostic disparities, drug access

## Abstract

Cystic fibrosis (CF) is an autosomal recessive disease caused by mutations in the cystic fibrosis transmembrane conductance regulator (*CFTR*) gene. Recently developed effective CFTR modulator drugs have substantially altered the disease trajectory of people with CF (pwCF) with access to these new therapies. Yet globally, this transformation to causal treatment remains profoundly unequal, with a substantial proportion of pathogenic *CFTR* variants in populations of non-European ancestry neither detectable by widely used genetic CF screening panels nor approved for or responsive to modulator therapies. This narrative review organizes the current global treatment realities along three axes: pwCF with access to CFTR modulator therapy; pwCF experiencing structural barriers in proper diagnosis and drug access; and those pwCF with biological realities not predisposing to currently approved effective CF drugs.

## 1. Introduction

**Figure 1:**
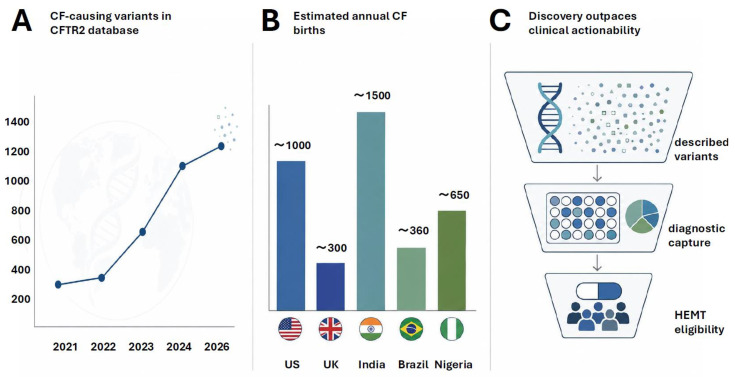
Expanding *CFTR* variant recognition, global birth burden, and actionability. (**A**) Number of CF-causing *CFTR* variants listed in CFTR2 across database updates. The list increased from 382 variants in September 2021 to 401 in April 2022, 719 in April 2023, 1085 in September 2024, and 1245 in January 2026, illustrating the ongoing expansion of clinically interpreted *CFTR* variation. (CFTR2 Variant List History/CFTR2 website updates. Updated January 2026. Available at: https://cftr2.org, accessed on 28 April 2026). (**B**) Estimated annual births with cystic fibrosis (CF) in selected countries. Although CF incidence per 100,000 births is highest in European-ancestry populations, high national birth rates substantially alter the absolute disease burden. India is estimated to have approximately 1426–1582 annual CF births, compared with approximately 1000 in the United States, 605–700 in Nigeria, 330–390 in Brazil, and approximately 300 in the United Kingdom (Bar et al., EBioMedicine. 2026) [5]. (**C**) Conceptual attrition from variant discovery to clinical actionability. Population gene sequencing identifies a broad and increasingly diverse set of pathogenic or potentially pathogenic *CFTR* variants; only a subset is captured by typical screening panels and diagnostic systems, and a further subset is currently approved for, or considered actionable with, highly effective modulator therapy (HEMT).

Cystic fibrosis (CF) is an autosomal recessive disorder caused by variants in the cystic fibrosis transmembrane conductance regulator (*CFTR*) gene. An increasing number of now more than 4000 *CFTR* gene variants have been reported, of which more than 1200 are confirmed disease-causing [1,2,3], see Figure 1A. The nature of the underlying *CFTR* variants determines the extent of protein dysfunction and consequently, the clinical phenotype: disease severity ranges from classic CF with meconium ileus, pancreatic insufficiency, and progressive respiratory disease to milder presentations with preserved pancreatic function and less lung involvement [3]. However, while the respiratory phenotype is not closely linked to the CF-causing gene defects, so-called milder *CFTR* mutations, associated with residual CFTR function and pancreatic sufficiency, for example, can lead to distinct clinical presentation and a delayed diagnosis [4].

**Figure 2:**
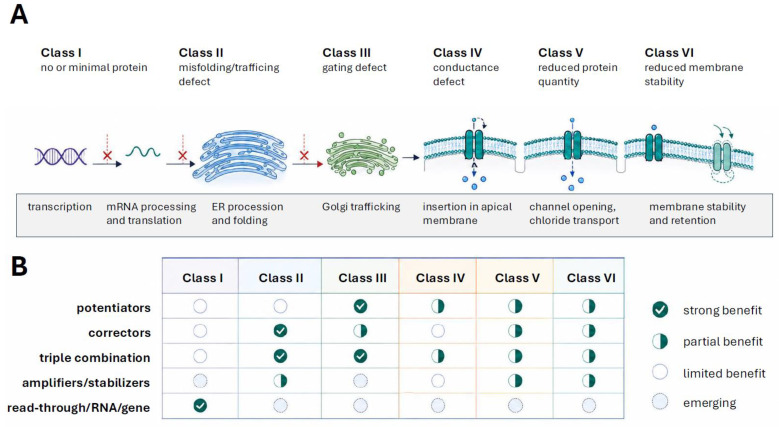
Classes of CFTR dysfunction and therapeutic options. (**A**) Simplified overview of major mechanistic classes of CFTR dysfunction along the CFTR biosynthetic and functional pathway. Class I mutations result in absent or minimal CFTR protein production; class II variants impair protein folding, processing, or trafficking; class III variants disrupt channel gating; class IV variants reduce ion conductance; class V variants reduce the amount of CFTR protein produced or inserted at the apical membrane; and class VI variants reduce membrane stability or retention. Class assignment is simplified as some variants produce more than one molecular defect. In (**B**), we show therapeutic strategies respective to mutation classes. Lightly filled dotted circles indicate emerging, conceptual, or investigational strategies. Potentiators primarily target gating defects and may benefit selected conductance or residual-function variants. Correctors and combination modulators are most relevant to folding, processing, or trafficking defects, particularly the F508del genotype, with additional variant-dependent effects. Amplifier or stabilizer approaches may increase protein quantity or membrane residence but remain context dependent. Read-through, RNA-based, gene-replacement, and gene-editing strategies are most relevant for variants that do not produce modulator-rescuable CFTR protein, including nonsense, frameshift, or large-deletion variants, but currently remain at early stages of clinical development.

For decades, CF care was predominantly symptomatic: nutritional optimization with pancreatic enzyme replacement, airway clearance physiotherapy, mucolytics, and inhaled or systemic antimicrobial therapies formed the backbone of clinical management. However, efforts towards understanding the consequences of the different gene mutation classes at the CFTR protein level then led to the development of specific CF drugs, Figure 2. The introduction of CFTR modulators—small molecules that restore CFTR protein expression and/or function at the cell membrane—such as the highly effective modulator therapies (HEMT) ivacaftor for people with CF (pwCF) and *CFTR* gating mutations (Class III) or elexacaftor/tezacaftor/ivacaftor (ETI) and vanzacaftor/tezacaftor/deutivacaftor (VTD) [6,7,8], initially approved for pwCF and at least one F508del mutation (Class II) was a major breakthrough and resulted in dramatic changes in a number of important outcomes in treated pwCF.

In well-resourced healthcare systems, HEMT has shifted CF from a disease of early or premature mortality to an increasingly manageable chronic condition in an aging population. In fact, pwCF who receive HEMT in high-income countries (HICs) now have projected survival modeled to extend into the seventh decade in some settings [9]. This therapeutic achievement, however, has simultaneously uncovered global disparities. Recent estimates suggest that only approximately 27% of the global CF population currently receives elexacaftor/tezacaftor/ivacaftor (ETI), with reimbursement of drug prices restricted almost exclusively to HICs [10]. Meanwhile, the majority of pwCF, particularly those in low- and middle-income countries (LMICs), remain undiagnosed, untreated, or both.

The global CF landscape is shaped by three major intersecting forces: First, the therapeutic evolution has delivered unprecedented clinical gains for those fortunate to have access to the new therapies (**The Good**). Second, structural barriers in proper diagnosis, CF registry coverage, regional health policies, and drug pricing exclude a majority of pwCF worldwide from the potential benefits of CFTR modulator therapy (**The Sad**). Third, the biological diversity of *CFTR* variants across populations of non-European ancestry imposes limits on the reach of current modulators, even if access barriers were removed; however, those may benefit from future progress in CF research (**The Hopeful**). These three axes are not necessarily without overlap; they interact in ways that compound inequity. Biology determines theoretical treatment eligibility, diagnosis determines whether a person is identified as having CF, and policy determines whether identified individuals can access available therapies. Inequity emerges from the intersection of all three.

This narrative review summarizes recent evidence across these three dimensions. We draw on large-scale genetic epidemiology from the Genome Aggregation Database (gnomAD) [5], global burden modeling [10], health technology assessments [11,12], real-world outcome data [13,14,15], and policy analyses to provide an integrated picture of CF care in the current modulator era. Although this is a narrative rather than a systematic review, we outline our approach to source selection for transparency. We searched PubMed and Google Scholar through May 2026 using combinations of the terms “cystic fibrosis,” “CFTR modulator,” “highly effective modulator therapy,” “access,” “low- and middle-income countries,” “newborn screening,” “*CFTR* variant,” and “health equity.” We prioritized peer-reviewed primary studies, large-scale genetic-epidemiology analyses, and national or international registry reports, supplemented by health technology assessments and regulatory documents (EMA, FDA) for current approval status. Given the rapidly evolving regulatory landscape, regulatory and registry sources were updated to the most recent available versions at the time of submission. Our aim is to move beyond a binary framing of CF as either “solved” or “neglected” and instead map the contours of a disease transformation that is real—but still unevenly distributed.

## 2. The Good: Therapeutic Transformation

### 2.1. Impact of HEMT in Clinical Trials and Real-World Practice

The introduction of HEMT marked a paradigm shift in CF care. In clinical trials, ivacaftor monotherapy for *CFTR* gating mutations [6], and the triple combination therapy ETI demonstrated meaningful improvements across multiple clinical endpoints: lung function, i.e., forced expiratory volume in one second (FEV_1_) increased by approximately 10–14 percentage points, the rate of pulmonary exacerbations (PEx) declined substantially, nutritional status and body mass index improved, and so did patient-reported quality of life [7,16].

Multiple real-world cohorts have meanwhile confirmed these treatment effects. A multicenter study using data from the German CF Registry, for example, reported sustained improvements in lung function and reductions in PEx frequency in the 12 months after ETI initiation [13]. Health claims–based analyses demonstrated a reduction in hospitalizations and healthcare utilization following the introduction of ETI [14]. Pediatric real-world data similarly confirmed significant improvements in pulmonary function and nutritional status with ETI in CF children and adolescents [15]. Findings were also confirmed in comprehensive health technology appraisals, including those conducted by Canada’s Drug Agency (CDA-AMC, formerly CADTH), which concluded that ETI provides substantial clinical benefit for eligible pwCF [12].

Vanzacaftor/tezacaftor/deutivacaftor (VTD), a more recently developed, once-daily CFTR modulator combination therapy, was approved by the US Food and Drug Administration (FDA) in December 2024 for pwCF aged 6 years and older carrying at least one F508del or another responsive mutation. The European Medicines Agency (EMA) approved VTD in mid-2025 for pwCF aged 6 years and older with at least one non-Class I *CFTR* variant [17].

In the SKYLINE phase 3 trials (VX20-121-102 and VX20-121-103), VTD demonstrated non-inferiority to ETI in pwCF aged 12 years and older with respect to change in percent predicted FEV_1_ through 24 weeks, with greater reduction in sweat chloride concentration [8]. The RIDGELINE trial extended these findings to children aged 6–11 years [18]. Now, both triple-combination therapies have been approved by the FDA for all mutations leading to CFTR protein production [19].

### 2.2. Redefining the Disease Trajectory

The therapeutic effects of HEMT are without doubt redefining what CF means for affected individuals and families, their health care providers, and also for the healthcare systems in those high-income countries with reimbursement policies covering the tremendous costs of the modulator drugs. In Europe, for example, while CF decades ago was predominantly seen as a pediatric disease, adults accounted for 55.5% of pwCF captured in patient registries in 2023. Recent reviews have emphasized that this epidemiologic shift in the age structure of CF populations is reshaping the provision of adult CF care [20,21,22,23]. Registry data from Europe, North America, and Australia document rising median survival, a growing adult CF population, and shifting patterns of morbidity [22,23]. The US Cystic Fibrosis Foundation (CFF) Patient Registry reported that among pwCF born between 2020 and 2024, half are predicted to live to age 65 or beyond [24]. The Cystic Fibrosis Canada (CFC) Registry predicted that half of babies born with CF in Canada in 2024 are expected to live beyond 64.3 years [25]. The German CF Registry reported 7771 people with CF in 2024, of whom 63% were adults, and a median projected survival age of 66.8 years for the 2020–2024 period [25]. Across Europe, the European Cystic Fibrosis Patient Registry (ECFSPR) documented 56,144 pwCF in 42 countries in 2023, of whom 55.5% were aged 18 years or older [20]. Although these registries employed period-based survival estimation methods, differences in cohort definitions, reporting periods, and population coverage mean that direct numerical comparisons of survival age estimates should be interpreted with caution.

### 2.3. Health-Economic Tensions

Despite the clinical benefits, HEMT poses significant health-economic challenges. Pharmacoeconomic analyses, including the CDA-AMC pharmacoeconomic review mentioned in Section 2.1, have reported very high incremental cost-effectiveness ratios (ICERs) for ETI at current pricing, exceeding conventional willingness-to-pay thresholds in most jurisdictions [11]. Annual therapy costs remain among the highest of any chronic disease, although manufacturing cost analyses have suggested that the minimum production cost of CFTR modulators is only a fraction of current market pricing [26]. The tension between clinical efficacy and cost-effectiveness is not merely an academic concern; it directly shapes access decisions in both HICs and LMICs. While cost may be a major, but not the sole determinant of access, as also shaped by reimbursement policies, regulatory pathways, and local health system capacity, these factors represent critical drivers of disparities as discussed in the following section.

## 3. The Sad: Structural Disparities in Diagnosis and Drug Access

The structural exclusion of most pwCF from the potential benefits of effective CFTR modulator therapies can be better understood as a causal chain with three links. First, the genetic burden of *CFTR* mutations and CF exists worldwide, including populations not traditionally recognized as CF-affected. Second, diagnostic capture fails in most LMICs due to the absence of newborn screening (NBS) for CF, limited laboratory infrastructure, and genetic screening panels that were designed for *CFTR* mutations common in populations of European ancestry. Third, even among properly diagnosed pwCF, access to HEMT is restricted by pricing, reimbursement policies, patent protections, and supply chain barriers. Each link compounds the previous one, and each is discussed in turn below. A note on terminology: throughout this review, we use genetic ancestry (e.g., European, African, South Asian) to refer to population-level patterns of CFTR variant distribution, as captured by genomic databases such as gnomAD. This is conceptually distinct from race and ethnicity, which are social constructs, and from geographic or national-income classifications (e.g., LMIC). These dimensions frequently correlate but are not interchangeable. Where we discuss disparities affecting specific racial or ethnic groups (e.g., Black Americans), we do so because the cited studies used those social categories, not as proxies for genetic ancestry.

### 3.1. The Global Prevalence of CF Is Larger than Recognized

A common and persistent misconception holds that CF is primarily a disease of White people with European ancestry. While CF is most common in Europeans (~1 in 2000–3000 births), evidence demonstrates, however, that it affects a much broader population than previously appreciated. Guo, King & Hill (2024) [10] modeled the global CF population at approximately 188,000, across 96 countries, of whom only about 112,000 (~59%) have been diagnosed. The remaining ~77,000 individuals are modeled to be undiagnosed, and are estimated to be overwhelmingly (82%) located in LMICs.

A complementary approach using the Genome Aggregation Database (gnomAD) has provided population-level estimates of CF incidence across different ancestral groups. Bar, Darrah & Vaidyanathan (2026) [5] estimated that CF affects 44–52 per 100,000 in European, 11–14 per 100,000 in Admixed American/Latino, 7 per 100,000 in African/African American, 6 per 100,000 in South Asian, 4 per 100,000 in Middle Eastern, and 0.2–1 per 100,000 births in East Asian populations, respectively.

### 3.2. Birth Rates Transform the Epidemiological Picture

Countries in South Asia, sub-Saharan Africa, and Latin America account for a large share of global births. When CF incidence rates are applied to actual regional birth statistics, the results are notable: India is estimated to have approximately 1426–1582 annual CF births, Nigeria 605–700, and Brazil 330–390, numbers comparable to the approximately 1000 annual CF births in the United States and 300 in the United Kingdom [5], Figure 1B. These figures are modeled estimates derived from applying ancestry-specific gnomAD-based incidence rates to regional birth statistics; they are not based on diagnosed-case surveillance and carry corresponding uncertainty, particularly given that gnomAD reflects general-population sequencing rather than confirmed CF cohorts. Country-level estimates thus suggest that annual CF births in selected high-birthrate LMICs are comparable to those in major high-income countries—continent-wide aggregation remains a data need [5].

Da Silva Filho et al. (2021) [27], reporting from the perspective of clinicians in Brazil, South Africa, Israel, and India, noted that CF remains associated with death in infants and young children in many LMIC settings, and that these countries may face significant challenges in promoting accurate CF diagnosis and improvements to CF care due to financial constraints and a significant burden of other diseases. It is therefore reasonable to infer that many children with CF in regions lacking diagnostic and therapeutic infrastructure experience high early mortality, though precise mortality data from undiagnosed populations are inherently unavailable.

### 3.3. Diagnostic Inequality

Early diagnosis is a cornerstone of effective CF management. Newborn screening (NBS) programs, which typically combine immunoreactive trypsinogen (IRT) and genetic testing, followed by confirmatory sweat chloride testing, have been implemented across much of Europe, Russia, North America, and Australasia [28,29,30]. However, NBS for CF is absent or incomplete in most LMICs. Da Silva Filho et al. (2021) [27] noted that the implementation of nationwide NBS programs in high-birthrate LMICs would require substantial investment and adaptation to local epidemiology and health system capacity.

Even where diagnostic capacity exists, it is often concentrated in a small number of tertiary centers [31]. The sweat chloride test, gold standard for CF diagnosis, requires standardized laboratory infrastructure that is not widely available in resource-limited settings [31]. Furthermore, as discussed in Section 4, genetic screening panels designed for populations with predominantly European ancestry systematically miss pathogenic variants more common in other populations, compounding diagnostic failure [5], Figure 1C.

### 3.4. Registry Gaps Impede Global Comparability

CF patient registries are essential tools for epidemiological surveillance, quality improvement, and outcome benchmarking. Robust CF registries exist in North America, Europe, and Australasia—the European Cystic Fibrosis Society Patient Registry (ECFSPR) alone captures over 56,000 pwCF from 42 countries [20]—but registry coverage in LMICs is sparse or nonexistent [32]. Without patient registries, the true CF prevalence, regional spectrums of *CFTR* variants, and the outcomes of available interventions remain unknown in most of the world. Naehrlich (2026) therefore argued that expanding global registry infrastructure is a prerequisite for meaningful international comparisons and for guiding resource allocation [32].

### 3.5. Access to HEMT Remains Largely Limited to High-Income Countries

Perhaps the most impressive manifestation of structural inequity in global CF care is in the distribution of access to CFTR modulators. Guo, King & Hill (2024) estimated that only approximately 51,000 of the 188,000 global pwCF (~27%) receive ETI [10]. ETI to date is reimbursed in 35 HICs but in only one LMIC in Europe—Bulgaria, a European Union member state classified as an upper-middle-income country by the World Bank at the time of the analysis [10], since reclassified as HIC in 2024. Even within the EU, access is not uniform: ECFSPR data from 2023 show that CFTR modulator uptake in several EU member states, including Lithuania and Estonia, remained among the lowest in Europe, comparable to non-EU countries without reimbursement agreements [20]. These intra-European disparities demonstrate that formal HIC classification does not guarantee equitable access to HEMT.

Among the approximately 112,000 diagnosed pwCF, roughly 14,000 live in countries where ETI was not available at all, four years after initial regulatory approval [10]. Da Silva Filho et al. (2021) noted that most individuals with CF in LMICs do not benefit from CFTR modulator therapy due to prohibitive costs [27]. However, the barriers to access are multifaceted. Manufacturing cost analyses demonstrated that the minimum production cost of CFTR modulators represents a small fraction of current prices, suggesting that substantial market price reductions would be economically feasible [26]. However, patent protections, the absence of generic competition in most markets, and the lack of tiered pricing agreements limit access.

In some LMICs, alternative access pathways have emerged, including court-based access, compassionate-use, or donation programs, locally produced or imported generics, and policy mechanisms such as compulsory licensing under TRIPS flexibilities [33,34]. Guo et al. (2025) described similar approaches in countries without reimbursement agreements, ranging from legal action to generic supply and donation programs [35]. However, these routes have generally benefited only small numbers of pwCF and do not provide a sustainable substitute for systematic pricing, reimbursement, and access strategies. While the discussion has rightly focused on HEMT such as ETI, access barriers also affect earlier-generation, double combination modulator drugs, i.e., lumacaftor/ivacaftor and tezacaftor/ivacaftor, which can represent the only available options for some pwCF based on genotype and age [36,37,38].

A systematic, peer-reviewed analysis of the CFTR modulator patent landscape across LMICs is lacking. Strategies to improve modulator access may be grouped by time horizon. In the short term, compassionate-use programs and price negotiations can reach limited numbers of patients. In the medium term, tiered (income-indexed) pricing, voluntary licensing agreements, and pooled regional procurement could broaden access more sustainably. In the long term, generic competition following patent expiry, compulsory licensing under TRIPS flexibilities, and local manufacturing capacity might offer the most durable route to affordability, alongside pharmacokinetic enhancement strategies that reduce per-patient drug requirements [39].

### 3.6. Outcome Disparities Between HICs and LMICs

Where comparative data exist, outcome disparities between HICs and LMICs are considerable. For example, a harmonized CF registries cohort analysis documented substantial differences in lung function, nutritional status, and survival between South African and Canadian pwCF in the pre-modulator era [40]. More recent data from South Africa illustrated the ongoing challenges of diversity and inequality in CF care in LMIC settings [41]. These disparities are expected to widen as HEMT becomes standard of care in HICs while remaining unavailable in most LMICs, though prospective data confirming the post-HEMT inequity trajectory are still emerging.

## 4. The Hopeful Horizon: Biological Constraints and Emerging Solutions

Before elucidating the biological constraints on CFTR modulator therapy, it is important to distinguish five distinct levels at which a person with CF may not benefit from modulator therapy (Table 1).

These categories are conceptually distinct but may overlap or co-occur in individual patients. Theratyping (Section 4.6) specifically addresses the gap between Levels 1 and 3.

### 4.1. F508del CFTR Is Less Common in Non-European Ancestry Populations

The development of modulators for the F508del *CFTR* variant was driven primarily by the needs of the European-ancestry CF population, in which F508del accounts for approximately 60–70% of the pathogenic alleles. In populations with non-European ancestry, F508del is substantially less prevalent. Population-level analysis of the Genome Aggregation Database (gnomAD) shows that F508del accounts for approximately 65% of all pathogenic *CFTR* alleles identified in individuals of European ancestry, but only 33% in Latino/Admixed American, 29% in African/African American, 32% in South Asian, 27% in Middle Eastern, and 9% in East Asian groups [5]. These figures reflect general population carrier data rather than diagnosed patient cohorts and may therefore differ from patient registry-based estimates, which are subject to ascertainment bias toward more easily diagnosed genotypes. Table 2 summarizes CF incidence, F508del frequency, the proportion of HEMT-ineligible alleles, and screening-panel coverage across ancestry groups.

### 4.2. A Substantial Fraction of Less Common CF-Causing CFTR Alleles Is Not Approved for Modulators

In addition to the lower frequency of F508del, a substantial proportion of pathogenic *CFTR* alleles in populations with non-European ancestry was not included in HEMT labels at the time of Bar et al.’s analysis [5]. In gnomAD v4, approximately 12% of European pathogenic alleles were not HEMT approved—already a recognizable gap. In non-European groups, this proportion rises to 30% in Latino/Admixed American, 40% in African/African American, 37% in South Asian, 28% in East Asian, and 60% in Middle Eastern populations [5].

An important distinction must be made between regulatory status and biological function. “Not HEMT-approved” means that a variant is not currently included within an approved label or reimbursement framework, but not that it is necessarily biologically non-responsive; this remains a moving target, as recently illustrated by the EMA’s non-Class I framework for vanzacaftor/tezacaftor/deutivacaftor and the April 2026 FDA label expansion to *CFTR* variants associated with protein production or demonstrated responsiveness. “Non-responsive” denotes that a variant produces a protein that is biologically unresponsive to available modulators, for instance, nonsense or frameshift variants that yield no functional CFTR protein. The overlap between these categories is substantial but not complete: some unapproved CF alleles may prove modulator-responsive upon functional testing, and it is this gap that theratyping (Section 4.6) aims to resolve. The proportions reported above, therefore, refer to regulatory ineligibility, not necessarily to biological non-responsiveness.

### 4.3. Screening Panels Miss Less Common Variants

The design of commonly used genetic screening panels contributes to diagnostic challenges. Most panels were developed based on *CFTR* variant spectrums in European ancestry populations and fail to capture variants prevalent in other population mixtures. Bar, Darrah & Vaidyanathan (2026) [5] evaluated four commonly used panels, i.e., Hologic (23 variants), EU2v1, Illumina (139 variants), and the expanded Wisconsin panel (>700 variants) against gnomAD data. Notably, the Hologic panel, one of the most widely used, misses 15% of European pathogenic alleles but 44% of Latino, 44% of African, 67% of South Asian, 74% of East Asian, and 68% of Middle Eastern alleles in gnomAD v4 [5]. Even the EU2v1 and Illumina panels miss about 25–55% of non-European *CFTR* alleles [5]. Only the expanded Wisconsin panel achieves reasonable sensitivity across groups, missing approximately 1–11% of alleles, but it contains over 700 variants and is not widely implemented [5]. This panel mismatch has direct clinical consequences: pwCF of non-European ancestry are more likely to receive false-negative screening results, leading to delayed or missed diagnosis even in countries where newborn screening (NBS) for CF exists. Comprehensive *CFTR* sequencing is a more sensitive alternative to mutation panels as it can identify rare coding, splice, and non-coding variants that panel-based strategies may miss. Recent reviews of CF NBS and molecular diagnostics suggest that whole-gene sequencing can improve diagnostic sensitivity and equity, although broader implementation remains more resource-intensive and depends on robust downstream variant interpretation [42].

### 4.4. Complex Alleles and Unpredictable Modulator Responses

Some *CFTR* alleles carry more than one variant in cis (complex alleles), which can alter protein folding, trafficking, and function in ways that are not predictable from individual variant data. Complex *CFTR* alleles may alter modulator responsiveness in unpredictable ways [43,44], as they may partially or completely abrogate the response to the modulator drugs, even when the individual component variants are theoretically responsive [45]. The prevalence and functional impact of complex alleles in populations of non-European ancestry are poorly characterized, representing another important knowledge gap.

### 4.5. Modulator Intolerance and Discontinuation

Among those pwCF who are eligible and have access to HEMT, there is documented variability in systemic drug exposures, and a subset experiences adverse events that necessitate dose reduction or discontinuation due to hepatotoxicity, neuropsychiatric, or other unwanted side effects [13,46]. Recent evidence suggests that pharmacokinetic variability may also contribute to suboptimal modulator response: concentrations of ETI components in blood are highly variable among treated individuals, and lower systemic drug exposures are associated with poor sweat chloride response, underscoring the potential need for personalized dosing strategies [47,48]. Systematic data on modulator discontinuation rates, adverse effect profiles, dose modification patterns, and pharmacokinetic variability across diverse populations are lacking.

Among those pwCF who are eligible and have access to HEMT, a subset experiences adverse events including hepatotoxicity and neuropsychiatric effects such as low mood, anxiety, sleep disturbance, and neurocognitive symptoms, which have been increasingly reported since ETI’s widespread adoption and that may necessitate dose reduction or discontinuation [49]. A systematic review of real-world data found that discontinuation and adverse-event rates may be higher than those observed in clinical trials, and identified a consistent mental-health and neurocognitive signal across all available CFTR modulators [50]. In some patients, individualized dose reduction can mitigate these effects while preserving clinical benefit [51], linking adverse-effect management to the broader question of pharmacokinetic variability: real-world German registry data document a range of systemic exposures and responses [13], and lower ETI component concentrations are associated with poorer sweat chloride response [47]. Pharmacokinetic variability is particularly relevant in children, in whom dosing and exposure differ from adults and remain incompletely characterized. Systematic data on discontinuation rates, adverse-effect profiles, dose modification, and pharmacokinetic variability across diverse and pediatric populations are still lacking.

### 4.6. The Residual Ineligible Population and Paths Toward Expanded Eligibility

Even in HICs with wide access to HEMT, a residual proportion of pwCF remains ineligible for modulator therapies. Desai et al. (2022) analyzed 2019 UK CF Registry data from 9887 individuals and found that 8.6% (*n* = 852) had no F508del allele and were therefore ineligible for elexacaftor/tezacaftor/ivacaftor under the then-current UK prescribing policy [52]. Registry-wide analyses using ECFSPR reported that approximately 13% of pwCF across 39 European countries were not eligible for CFTR modulators in 2021, with a substantially higher proportion in Mediterranean countries (Turkey 43.5%, Israel 41.2%, Italy 26.2%) [conference abstract] [53]. The German CF Registry similarly documented that F508del accounted for 66.3% of all CF alleles, with CFTR modulator therapy available for 88% of the registered population [25]. In ECFSPR, the F508del allelic frequency varies considerably across European countries, with the highest frequency in Denmark (81.9%) and the lowest in countries bordering the Middle East and Caucasus [20]. Other regionally enriched modulator-responsive variants, such as G551D in Ireland, further illustrate that European *CFTR* variation is not homogeneous. In populations with non-European ancestry, the ineligibility rate is expected to be substantially higher, given the lower frequency of HEMT-approved variants, as already discussed in Section 4.2.

Recognizing that many pwCF carry *CFTR* variants not included in current HEMT labels, there is growing interest in theratyping, the functional characterization of individual variants to determine responsiveness to modulators. In vitro studies have evaluated modulator responses for hundreds of *CFTR* variants, identifying subsets that respond favorably despite not being formally approved [54]. Regulatory pathways for expanding HEMT labels based on theratyping data are being developed, but progress is slow and resource-intensive.

Theratyping is an important mechanism that can narrow the gap between “not approved” and “non-responsive” identified in Section 4.2. As of April 2026, the recent EMA authorization of vanzacaftor/tezacaftor/deutivacaftor (VTD) for pwCF aged 6 years and older who carry at least one non-Class I *CFTR* mutation, together with the April 2026 FDA expansion to patients with at least one *CFTR* variant that is either responsive or results in production of CFTR protein, signals a regulatory shift toward a broader protein-based eligibility framework beyond traditional variant-by-variant approval. If extended globally, such an approach could substantially reduce the need for individual variant theratyping and expand the treatable population, though it would also raise questions about the cost implications of widening eligibility in resource-constrained settings. To date, theratyping efforts have, however, predominantly focused on variants common in populations with European and North American ancestry. Extending these efforts to variants prevalent in diverse, South Asian, African, Middle Eastern, and East Asian populations remains a critical unmet need [45,54].

### 4.7. Novel Approaches to Treat CF Beyond Modulators

For pwCF and *CFTR* variants not producing a protein amenable to modulator rescue, particularly those with nonsense, frameshift, or large deletion mutations, alternative therapeutic strategies are needed, Figure 2. Consequently, several promising approaches are in preclinical or early clinical development.

Gene replacement therapies, which are mostly targeting the airways, aim to deliver a functional copy of the *CFTR* gene directly to the airway epithelium. Despite decades of effort, efficient and durable transfection of epithelial cells remains challenging, primarily due to barriers posed by airway secretions, immune responses to viral vectors, and the need for repeated dosing to overcome low transfection rates and epithelial cell turnover [55]. Alternative mRNA-based, agnostic therapies represent a potentially more tractable approach: inhaled CFTR mRNA delivered via lipid nanoparticles, for instance, could restore CFTR protein expression regardless of the underlying variant class. A phase 1/2 trial of inhaled CFTR mRNA has shown safety and tolerability, though clinical efficacy data remain limited [56]. CRISPR/Cas9-based gene editing in airway stem cells has demonstrated proof-of-concept correction of CFTR function in patient-derived cells [57,58], but translation to in vivo therapy faces substantial hurdles. Delivery systems based on the helper-dependent adenoviral (HD-Ad) vector, for example, capable of delivering genes to airway basal stem cells in vivo, have been shown to succeed in co-delivery of both CRISPR-Cas9/single-guide RNA and the LacZ reporter or *CFTR* gene as donor DNA to cultured cells [58]. For nonsense mutations specifically, translational readthrough agents that suppress premature stop codons are under investigation, with early-stage compounds showing restored CFTR function in vitro [59].

These mutation-agnostic or class-specific strategies are of particular relevance to CF populations with non-European ancestry, where the proportion of modulator-unresponsive variants is highest. However, none of these alternative approaches has yet advanced to late-stage clinical development, and timelines for regulatory approval remain uncertain. The CFF therapeutic pipeline currently lists over a dozen gene therapy programs (including AAV-based gene therapy, multiple inhaled mRNA candidates, gene editing approaches, and antisense oligonucleotides) alongside novel readthrough compounds, with several in phase 1–2 trials [60]. However, the path from early clinical development to globally accessible therapy will require sustained investment, international trial participation, and deliberate inclusion of diverse *CFTR* genotypes in study designs.

Beyond specific therapeutic modalities, artificial intelligence (AI) and machine learning (ML) are increasingly being applied across the CF therapeutic development pipeline. In drug development, AI/ML methods are being used to accelerate the identification of candidate compounds and to optimize inhaled formulations and pulmonary drug delivery systems [61]. In parallel, AI-driven analysis of thoracic imaging offers a route to more sensitive, individualized assessment of treatment response—potentially including the identification of likely responders among patients not currently eligible for modulator therapy, complementing in vitro theratyping approaches [62].

While these approaches are scientifically promising, none has yet been established as a proven substitute for CFTR modulators in routine clinical care. Even gene therapy, under investigation for over two decades, has not achieved durable clinical translation. For the foreseeable future, CFTR modulators remain the only highly effective disease-modifying therapy in routine use, and expanding equitable access to them remains the more immediate global priority.

## 5. Intersections: Where Biology, Diagnosis, and Policy Converge

**Figure 3:**
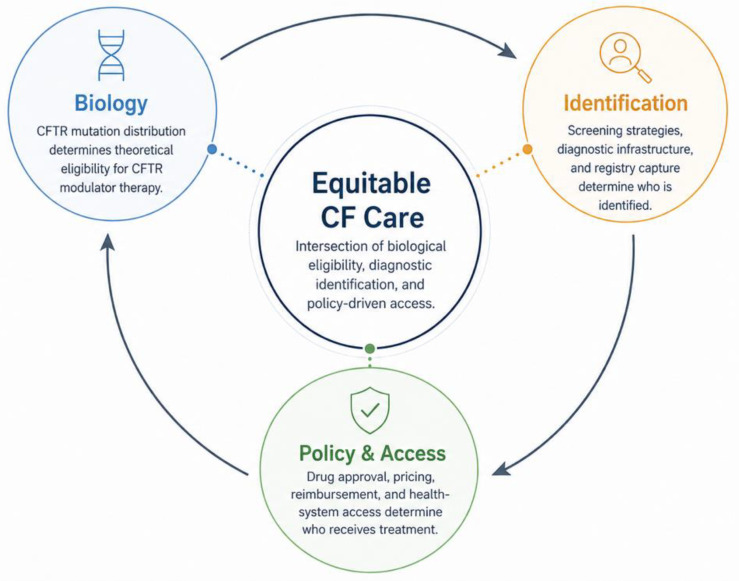
Framework for inequity in the current CFTR modulator era.

The three axes described above do not operate in isolation; they interact in ways that compound inequity and create a landscape of overlapping disadvantage. Figure 3.

Equitable CF care depends on the intersection of three interacting domains. **Biology** determines theoretical treatment eligibility through the underlying *CFTR* genotype and its molecular responsiveness to available or emerging therapies. **Identification** determines whether a person with CF is detected through newborn screening, diagnostic infrastructure, sweat chloride testing, sequencing strategy, and registry capture. **Policy and access** determine whether an identified and biologically eligible person can receive treatment through drug approval, pricing, reimbursement, supply chains, and health-system capacity.

Biology determines theoretical drug eligibility. A person’s CFTR genotype determines whether currently available modifier therapies could, in principle, improve their health. Populations with non-European ancestry carry a higher proportion of CF alleles that are not HEMT-approved or -responsive, narrowing the pool of treatable individuals. The experience of Black Americans with CF, for instance, can be used to illustrate how these axes compound within a single healthcare system. Black pwCF carry a higher proportion of CFTR variants not responding to HEMT, reducing their biological eligibility. At the same time, socioeconomic disadvantage limits access to specialized care and modulator drugs. US transplant registry data had shown that since the introduction of HEMT, the proportion of non-White pwCF among lung transplant candidates had increased, likely because White pwCF on average benefited from HEMT, whereas Black and Hispanic pwCF as a group did not, as they more often carry CFTR variants not responsive to currently available modulators [63]. These biological eligibility gaps intersect with broader access barriers: in a matched cohort linking the CFF Patient Registry with U.S. transplant registry data, lower neighborhood socioeconomic position was independently associated with reduced waitlisting for lung transplantation, even after adjustment for markers of disease severity [64]. Earlier diagnosis through equitable newborn screening and expanded modulator eligibility through theratyping would potentially interrupt this vicious circle—but only if accompanied by deliberate efforts to address the social determinants of access to appropriate care.

Diagnosis determines identification. Even a person with a HEMT-responsive genotype cannot benefit from treatment if not identified as having CF. Screening panels that miss less common variants, combined with the absence of NBS infrastructure in most LMICs, ensure that a large proportion of potentially treatable pwCF is never diagnosed properly.

Policy determines access to effective therapy. Among those who are both genetically eligible and diagnosed, access to HEMT depends on regulatory approval, reimbursement decisions, pricing, and supply chain logistics. These factors are overwhelmingly favorable in HICs and overwhelmingly unfavorable in LMICs.

The intersection of these three domains means that a child born with CF in India, Nigeria, or Brazil for example, faces a cascade of compounding disadvantages: their *CFTR* genotype is less likely to be detectable by standard screening panels, less likely to be diagnosed in the absence of NBS, less likely to be eligible for HEMT, and, even if eligible, less likely to have access to therapy, Figure 3. Each barrier independently reduces the probability of receiving effective treatment; together, they produce profound exclusion from the potential benefits of the modulator era for many pwCF in LMICs.

This framework also highlights the importance of an integrated response. Improving any single domain in isolation, for example, expanding drug access without improving diagnosis, or improving diagnosis without addressing biological eligibility, will yield limited gains. A globally inclusive CF strategy must address all three axes simultaneously.

## 6. Discussion

### 6.1. Beyond Binary Narratives

The current modulator era invites a binary narrative: CF is either “cured” or it is not. Neither framing is accurate, and both are harmful. The “cure” narrative is inaccurate on two counts: most pwCF worldwide do currently not benefit from modulator drugs, and those who do are not cured, but continue to show residual disease and ongoing progression [65,66]. It may also diminish support for continued novel therapeutic developments, particularly for variants unresponsive to current modulators. Conversely, dismissing the modulator evolution disregards the profound and measurable improvements in quality and length of life experienced by many successfully treated pwCF.

The three-axis framework proposed here—The Good, The Sad, and The Hopeful—is intended to hold these realities simultaneously. The therapeutic transformation is a reality. The structural exclusion of most of the world’s pwCF from its benefits is also real. And the biological limits of current therapies are an orthogonal constraint that no amount of policy reform alone can overcome.

### 6.2. Ethical Dimensions

The current distribution of HEMT access raises fundamental questions of distributive justice. A child born with CF and HEMT-responsive genotype in North America or Europe can expect projected survival extending well into adulthood with maintained lung function. A child born with the same genotype in India or Nigeria, for example, is likely to face missed or late diagnosis, absence of effective treatment, and substantially shortened survival. This disparity is not an inevitable consequence of biology; it reflects differences in drug pricing, reimbursement structures, regulatory pathways, and investment in global health infrastructure [33,34].

The ethical imperative extends beyond access to existing therapies. The concentration of genomic research, variant characterization, and therapeutic development populations with European ancestry means that pwCF with non-European ancestry face not only access barriers but also knowledge gaps: less is known about their disease phenotypes, their genetic variants, and their potential responses to treatment. This represents a form of structural epistemic inequity that perpetuates clinical disadvantage [5,67].

### 6.3. Toward Globally Inclusive CF Care

A globally inclusive CF strategy will require action across multiple domains simultaneously. On the diagnostic front, the declining cost of next-generation sequencing may make comprehensive *CFTR* gene analysis increasingly feasible, particularly in settings where the local variant spectrum is poorly characterized [5]. Full-*CFTR* sequencing could serve as a universal diagnostic tool, circumventing the limitations of *CFTR* mutation panels.

On the therapeutic front, expanding access to HEMT through pricing reform would be a reasonable goal. However, for the substantial proportion of pwCF with non-European ancestry carrying HEMT-ineligible variants, alternative therapeutic strategies are urgently needed. Nonsense variant readthrough agents, mRNA therapies, and gene replacement and editing approaches offer the prospect of mutation-agnostic treatments that could bypass the genotype-specific limitations of current modulators [56,57,59].

On the policy front, the establishment of global CF registries, development of LMIC-adapted clinical guidelines, and investment in local diagnostic and treatment capacity are essential foundations for equitable care [27,31,32].

Taken together, responsibility for action must be distributed across all stakeholders. Clinicians can advocate for equitable screening and document outcomes in underrepresented groups. Researchers should prioritize functional characterization of non-European variants and inclusion of diverse CFTR variants in trials. Registries must expand into underserved regions to enable benchmarking. Regulators can advance protein-based, theratyping-informed eligibility frameworks. Payers and governments determine reimbursement and can pursue tiered pricing and pooled procurement. Industry holds the levers of pricing, licensing, and trial design. Patient organizations sustain advocacy and awareness. Progress requires coordinated, simultaneous action across all of these.

### 6.4. Limitations

Several limitations should be acknowledged. This narrative review does not apply a systematic search or quality-appraisal methodology and is subject to selection bias in the cited literature. Much of the global epidemiological picture rests on modeled estimates rather than diagnosed-case surveillance, particularly for LMIC populations where registry data are sparse or absent. Genetic-epidemiology figures derived from gnomAD reflect general-population sequencing and are subject to ascertainment bias. The regulatory landscape is changing rapidly, and approval statuses cited here may evolve. Finally, functional (theratyping) data remain limited for many rare *CFTR* variants, especially those prevalent in non-European populations, constraining firm conclusions about their modulator responsiveness.

## 7. Conclusions

The CFTR modulator era represents one of the most remarkable therapeutic transformations in the history of individualized precision medicine. For pwCF in well-resourced settings with HEMT-responsive genotypes, the disease trajectory has substantially improved. However, this transformation advances profoundly unevenly. Only approximately one quarter of the global CF population currently receives effective modulator therapy. The majority of pwCF worldwide remain undiagnosed, untreated, or ineligible for effective drugs due to the convergence of structural barriers and biological limitations.

Achieving global equity in CF care will require an integrated strategy that addresses all three axes identified in this review. Access to effective therapy must be expanded through pricing reform and policy innovation. Diagnostic capacity must be strengthened through population-appropriate screening strategies, including expanded panels or full-*CFTR* sequencing. The biological knowledge gap must be closed through the systematic characterization of *CFTR* variants in populations with non-European ancestry and the development of mutation-agnostic therapeutic approaches to restore CFTR function.

The good news is real. The sad reality is urgent. The unfortunate biological constraints are hopefully addressable. Ensuring that the modulator era benefits not just a fortunate minority globally, but all people living with cystic fibrosis, will require coordinated action across research, diagnostics, policy, and access frameworks.

## Figures and Tables

**Table 1:** Five levels at which a person with CF may not benefit from CFTR modulator therapy.

Level	Category	Definition	Illustrative Example
1	Biologically non-responsive	The *CFTR* variant produces a protein (or no protein) that does not respond to currently available modulators	Nonsense or frameshift variants yielding no functional CFTR protein
2	Not yet functionally tested	The variant has not been characterized in vitro or in vivo for modulator responsiveness	Rare variants absent from theratyping datasets
3	Not currently label-approved	The variant is not included on regulatory drug labels, regardless of potential biological responsiveness	A potentially responsive variant not yet reflected in current regulatory labeling
4	Not accessible in practice	The drug is approved for the patient’s genotype but unavailable due to pricing, reimbursement, or supply-chain barriers	Eligible pwCF in an LMIC without reimbursement
5	Intolerance	The patient is eligible and has access but cannot start or continue therapy due to adverse effects	Discontinuation due to hepatotoxicity or neuropsychiatric side effects

**Table 2:** CF incidence, F508del frequency, source-framework HEMT non-approval, and limited-panel miss rate by genetic ancestry group.

Genetic Ancestry Group	Estimated CF Incidence per 100,000 Births ^1^	F508del (% of Pathogenic Alleles) ^1,2^	Pathogenic Alleles Not HEMT-Approved in Source-Analysis Framework (%) ^1,3^	Hologic Panel Miss Rate (%) ^1,4^
European	44–52	~65	~12	15
Admixed American/Latino	11–14	~33	~30	44
African/African American	7	~29	~40	44
South Asian	6	~32	~37	67
Middle Eastern	4	~27	~60	68
East Asian	0.2–1	~9	~28	74

^1^ Incidence, F508del allele-frequency, source-framework HEMT non-approval, and Hologic panel miss-rate estimates are derived from the gnomAD v2.1 and v4 population-genomic analysis by Bar, Darrah & Vaidyanathan (2026) [5]. These estimates are based on general-population carrier data and Hardy–Weinberg modeling, not on diagnosed CF registry cohorts. ^2^ F508del percentages refer to the proportion of pathogenic *CFTR* alleles in the source analysis. They should not be confused with registry-derived patient-level proportions such as “at least one F508del variant,” which use a different denominator and are affected by diagnosis, ascertainment, and registry participation. ^3^ “Not HEMT-approved” reflects the regulatory approval framework applied in the source analysis. This column should not be interpreted as current 2026 treatment ineligibility or as biological non-responsiveness. HEMT eligibility is jurisdiction-, label-, and date-dependent and has expanded substantially in recent US and EU regulatory updates. ^4^ The Hologic panel miss rate is retained as a source-defined comparator for limited-panel screening. It illustrates ancestry-dependent residual risk under historically restricted variant panels and should not be interpreted as the performance of contemporary expanded *CFTR* sequencing or large variant panels. CFTR2, registry reports, and regulatory labels were used as contextual sources for *CFTR* variant classification, diagnosed-cohort composition, newborn-screening panel interpretation, and current modulator-label context. They were not used to overwrite the ancestry-specific numerical estimates in Table 2, which are derived from the Bar, Darrah & Vaidyanathan gnomAD analysis [5].

## Data Availability

No new data were created or analyzed in this study. Data sharing is not applicable to this article.
